# An emerging framework for fully incorporating public involvement (PI) into patient-reported outcome measures (PROMs)

**DOI:** 10.1186/s41687-019-0172-8

**Published:** 2020-01-13

**Authors:** J. Carlton, T. Peasgood, S. Khan, R. Barber, J. Bostock, A. D. Keetharuth

**Affiliations:** 1 University of Sheffield, Regent Court, 30 Regent Street, Sheffield, S1 4DA UK; 2 University of Cambridge, Cambridge, UK; 3 University of Oxford, Oxford, UK; 4 Kings College London, London, UK

**Keywords:** Public involvement (PI), Patient and Public Involvement (PPI), Patient reported outcome measures (PROMs), Questionnaire

## Abstract

Patient-reported outcome measures (PROMs) are widely used in the United Kingdom (UK) and internationally to report and monitor patients’ subjective assessments of their symptoms and functional status and also their quality of life. Whilst the importance of involving the public in PROM development to increase the quality of the developed PROM has been highlighted this practice is not widespread. There is a lack of guidance on how public involvement (PI) could be embedded in the development of PROMs, where the roles can be more complex than in other types of research. This paper provides a timely review and sets out an emerging framework for fully incorporating PI into PROM development.

## Introduction

Patient-reported outcome measures (PROMs) are widely used in the United Kingdom (UK) and internationally to report and monitor patients’ subjective assessments of their health. PROMs can be used to measure symptoms, functional status and quality of life. PROMs were initially developed for use in research to assess the effectiveness of treatments however they are now also used to assess and compare the outcomes achieved by healthcare providers [1]. Additionally, PROMs may be used for information sharing and to support patient decision-making [2]. The value of incorporating patient input and the patient’s own perspective on their own quality of life when designing new PROMs is now almost universally accepted. PROM developers have moved away from the historical “top down” methods to “bottom up” methodologies, which incorporate qualitative data techniques to generate content for the PROM itself [3]. Such changes have improved the content validity of PROMs, ensuring that the language and terminology are appropriate for the target population, and the items within the instrument fully assess the impact of the given condition on quality of life (for example). The Food and Drug Administration (FDA) guidance on PROM development advocates patient input to inform the content and improve the relevance and validity of PROMs [4]. Whilst the guidance advocates the important role of patient input, it could be argued that this can be met by having patients or members of the public as research participants in the early stages of PROM development. However, the value of using patients or members of the public to advise on the *process* of PROM development and help guide the research is less acknowledged.

There is growing recognition of the value of public involvement (PI) in health research, which has been defined as “research being carried out with or by members of the public” [5]. In this context, the term “public” includes patients, potential patients, carers and people who use health and social care services, as well as organisations that represent people who use these services [5]. The use of the term Public Involvement (PI) is preferred over Patient and Public Involvement (PPI). With the release of the National Standards for Public Involvement and the focus on inclusivity, using the term PI is seen as more reflective of the standards and the fact public involvement is not limited to just patients but includes carers and members of all communities in research [6]. Different terms are used in other countries, such as ‘consumer involvement’ or ‘public engagement’ [7].

Including the public as advisors (rather than participants) in research has become increasingly embedded within health and social care research. Guidance and standards are available to support researchers in achieving effective public involvement [5, 6]. Members of the public may contribute, for example, by commenting on study design, informing the selection of outcome measures, reviewing patient information sheets and consent procedures, making suggestions about data collection methods and advising on follow up processes [8]. The incorporation of PI into healthcare research is part of a family of participatory research methods which aim to transfer power from the researcher to the research participant giving them control of the research agenda, the process and future actions [9]. Within PROM development, PI in has allowed individuals to be included as part of a research team. By its definition, PI is research being carried out ‘with’ or ‘by’ members of the public, rather than ‘to’, ‘about’ or ‘for’ them [10]. There can be differences between the perspectives of researchers and those who expect to benefit from research (i.e. patients) on important aspects of the research process, such as the relevance of outcome measures [11, 12]. PI brings a “unique perspective” which enriches research [13, 14].

The importance of involving the public in PROM development to increase the quality of the developed PROM has been highlighted by many authors [12, 15, 16]. PI and public engagement in PROM development and/or health outcomes research is encouraged by a range of institutions [17, 18], however this practice is not widespread. A recent systematic review of the international literature reported that a quarter of the 193 PROMs (*n* = 50) included in the review did not include any PI in their development [19]. There was minimal involvement in the selection of outcomes to measure within the PROM (10.9%), and very few cases with involvement in all stages of the PROM development [19]. However, it should be noted that there are discrepancies in terminology between what some researchers refer to as PI and patients as research participants [20]. PI is not the same as patient participation. There have been calls for clarity, guidance and consensus on PI in PROM development [13, 15]. It is clear that more collaborative PI needs to become the norm throughout all stages of co-construction, selection and implementation of PROMs [21]. Previous studies have demonstrated PI has benefits in increasing content validity of PROMs, however there is limited guidance as to what to consider when developing a new instrument. This paper provides a timely review and sets out an emerging framework for fully incorporating public involvement into PROM development. The content is based upon our own experiences as researchers working with public representatives in PROM development, rather than a systematic review of the empirical evidence. We do not intend to be prescriptive rather offer a framework to serve as a prompt and reference point of stages to consider including PI when developing a PROM. In the first section, we provide a guide of where to embed PI in PROM development. The second section is a discussion of the challenges of PI in PROM development.

### Public involvement and patient-reported outcome measures

**Fig. 1:**
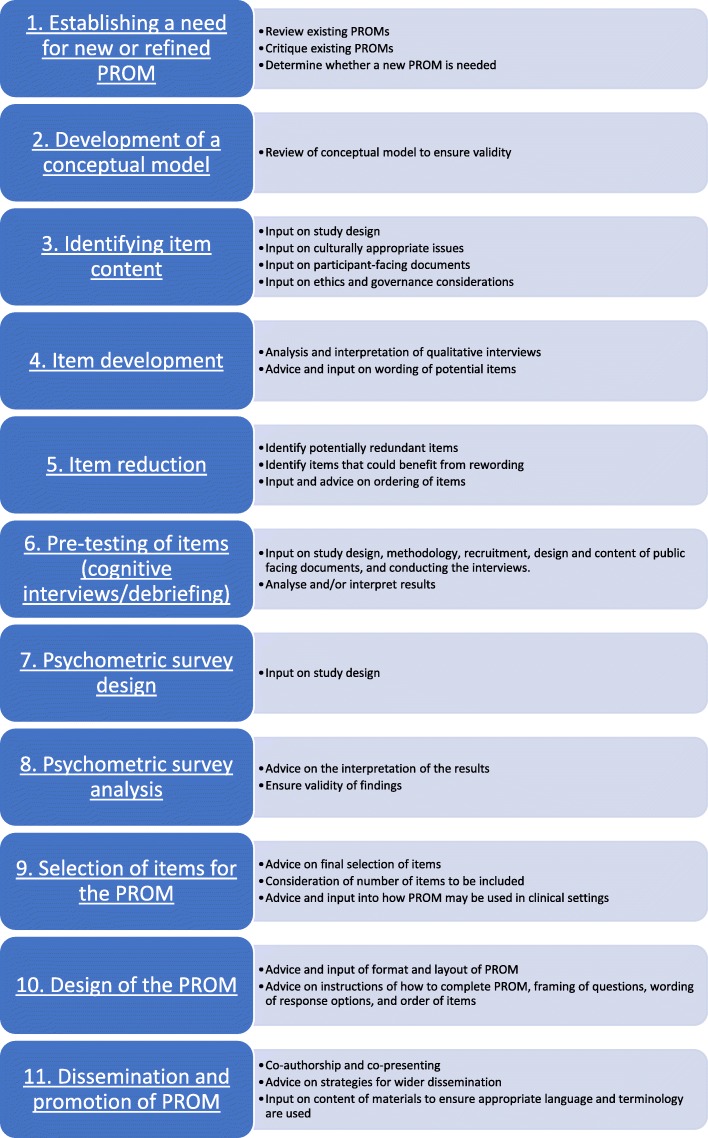
Public involvement in different stages of PROM development

There are potential difficulties in identifying where PI can occur in the development of a PROM and in the application of the range of research methods used. There are many stages of PROM development (as outlined below) that could be followed; however, these are not always undertaken either due to time or financial constraints. There are also cases where the target population for which the PROM is intended may not lend itself to involvement in all the stages of PROM development outlined here (such as in rare health conditions, as discussed later). We have identified where PI could occur at each stage of PROM development. However, time and financial limitations may mean that PI cannot be fully incorporated throughout. Across all stages of the primary research, PI can assist with understanding and interpreting the study findings [22]. The stages described here identify potential steps that can be undertaken when developing or refining a PROM. They are not meant to be prescriptive, and the process may not always be linear. It should also be noted that PI may involve input from more than one individual. Involving more than one person increases the breadth of experience to the project, allows those involved to support and encourage each other, and allows for multiple perspectives. PI members cannot be representative of everyone who has a specific condition [23, 24]. Having a PI team is beneficial to allow for wider diversity and experiences. The stages are summarised in Fig. 1.

#### Establishing a need for a new or refined PROM

The first stage in which PI may occur is when considering if a new PROM is necessary. There are many examples of PI instigating research in key areas, and initiatives such as the James Lind Alliance [25] in the UK and Patient-Centred Outcomes Research Institute [26] have facilitated joint partnerships with patients and clinicians to prioritise treatment uncertainties for research. PI can be useful in reviewing the quality of existing PROMs, critiquing them in terms of content and applicability, and suggesting whether a new measure should be developed [12, 21].

#### Development of a conceptual model

If a new or refined PROM is required, researchers may devise a conceptual model for the PROM: the specific measurement goal and the domains or themes that the questionnaire should cover. The conceptual model may be informed by existing literature and clinical opinion, however PI can be incorporated to ensure validity and provide a sense check of the literature [21].

#### Identifying item content

Traditionally the content of PROMs was informed by the literature and/or clinical opinion [27]. Over recent years there has been an increasing commitment to identify potential items (questions) and themes through qualitative methods with the target population. Interviews or focus groups with people for whom the PROM is intended can be conducted to identify exactly how that health condition impacts upon an individual’s quality of life. The transcripts of the qualitative work can be analysed to identify themes that can be matched to the conceptual model (which helps to validate the model) or used to refine the model. Furthermore, the language used by the participants can be used later in Stage 4 to phrase potential items to be included in the PROM. This stage typically involves patients as research participants and would not be considered PI. However, at any stage in which patients (or the public) are included as research participants there is also an important role for PI in providing advice. PI involvement ensures that the best data is generated to support the PROM development and helps mitigate against some of the ethical issues that surround measure development. The key role for PI in Stage 3 is ensuring it is relevant to potential research participants and carried out in a way that is right for them. PI can occur in several aspects of Stage 3. Firstly, in designing the study, PI can identify who would be the most important group of people to include in the qualitative work (e.g. patients, carers, clinicians, general population). If patients are the target population, PI input may include establishing inclusion and exclusion criteria (i.e. which patients to include in qualitative data collection). Advice on what would be the most appropriate data collection method (i.e. interviews vs. focus groups), the timing of interviews, the location, and on who should conduct the interviews can all be incorporated [16, 28]. The most appropriate data collection method may differ from one perspective to another – whilst focus groups can be more efficient and cost-effective than interviews, they are not suitable for every topic. Peer (or lay) interviewing has been used in PROM development [16]. Furthermore, input on the content of topic guides, information sheets, consent forms, and all other patient-facing documents and guidance on any ethical implications of the study (including potential risks) are all key areas for PI. The PI partners may be able to provide their own contacts to help with recruitment, such as links with charities, support groups and/or referrals to community resources.

#### Item development

Stage 4 involves taking the results of Stage 3 and wording the quality of life (QoL) concepts and issues into potential items to be included in the PROM or identifying existing items that capture the concept from item banks (repository of items) or existing measures. PI can further ensure the validity of the PROM through analysis and interpretation of the qualitative data alongside other members of the research team. Advice can be given on the wording of the potential items to ensure that lay language is used. The wording of items could come either from PI members or from the transcripts of patients as research participants (in which case PI can occur as noted in Stage 3). [The advantages of PI input rather than that of research participants will concern time and their broader understanding of the overall project.] Stage 4 will generate a long list of potential items. PI may also identify any items which could be upsetting or offensive within the context of the health condition. There is also potential for PI to help with supporting translation and cultural adaptation for use in other countries. For example, PI could identify any potential difficulties with translating particular items to other languages if they themselves are bilingual, or by identifying items which may be problematic or challenging in different cultures.

#### Item reduction

In some cases, Stage 4 will generate a list of potential items that may be too onerous if they were all to be included in a piloting or cognitive debriefing stage (Stage 6). PI can be part of a collaborative exercise to develop the criteria used to identify suitable items and to judge whether the items meet the criteria. PI can also identify any potentially redundant items, or indeed reword items to improve clarity. Advice may also be given concerning the ordering of the items for Stage 6. This is an area that can often be overlooked in PROM development. Consideration should be given to the implications of the potential impact of particular questions to the person completing the questionnaire, as well as the ordering of the questions. The ordering of the questions in Stage 5 does not necessarily determine the ordering of questions within the final PROM.

PI can shed light on how it would feel to be the patient or individual to experience completing a questionnaire by commenting on potentially negative questions (which might make people focus on negative aspects of their life), positive questions (which might be upsetting if they appear insensitive), and items which might seem irrelevant (which might be frustrating). The explanation and ordering of the questions should be carefully considered [29].

#### Pre-testing of items (cognitive interviews and debriefing)

The aim of Stage 6 is to refine and pre-test items before a larger psychometric survey. PI allows for items, response options, and instructions for questionnaire completion to be considered. As with Stage 3, PI may include input on study design and methodology, recruitment, design and content of the public facing documents, and conducting the interviews themselves [16]. Further PI may involve analysing or interpreting the results of the interviews to select the items to include in the psychometric survey (Stage 7).

#### Psychometric survey design

The aim of this stage is to test the performance of potential items when completed by the target population. PI can be useful in Stage 7, particularly with respect to the proposed methods of data collection. Advice and guidance can be given in a number of key areas: the identification of potential participants; mode of survey administration; public facing documentation; appropriateness of additional information required for comparison and validation, including other PROMs and other questions that could be asked of the respondent to identify the severity of their health condition; response burden for potential participants; and the appropriateness and method(s) of incentivisation. More creative methods of mode of administration may need to be considered if the PROM is for particular populations (such as those with language difficulties, learning difficulties and dementia) [30]. PI can consider the limitations and/or specific needs of participants, their suitability for the study, eligibility criteria, potential ethical issues related to participating in the psychometric survey, administration and recruitment.

#### Psychometric survey analysis

PI in this phase of PROM development can be useful for the interpretation of results. The different perspectives offered by PI partners may identify issues missed by researchers, and help to ensure the validity of the findings from the patient and/or public perspective [16].

#### Selection of items for the PROM

The final item selection can be informed by combining information from different sources, such as the results of the cognitive interviews, the psychometric survey, clinician input, and PI [16]. It is crucial to have PI in Stage 9 to ensure that items are selected not only on their performance, but also on their suitability for the target population. The number of items to be selected can also be informed by PI, taking into account the potential response burden for those for whom the PROM is designed, and also how the PROM may be used in routine clinical care.

#### Design of the PROM (including layout and response options)

How a PROM is presented (either on paper or online) is often an under-reported component of PROM development. PI in Stage 10 is important, so that key issues such as the format and layout of the questionnaire are designed to be appropriate for the target population. As with Stages 4, 5 and 6, advice can be given on instructions on how to complete the questionnaire, suitable wording of response options, framing of the questions (such as yesterday, last week, last month) and the order of the items. The order of items in the final questionnaire is informed by a number of sources, which may include PI input, clinical opinion and an understanding of order effects [31].

#### Dissemination and promotion of the PROM

PI in Stage 11 can engage with the general dissemination of the measure and, more specifically, promote the role PI had in its development. Activities such as co-authorship, co-presenting to various audiences (such as academics, clinicians, social care professionals, patient groups and lay audiences), and advising on strategies for wider dissemination should be encouraged. PI members can help to ensure materials are written in lay language. Endorsements from PI participants can help reassure potential users of the PROM that their voices have been heard during the development process. This approach was recently adopted in the development and implementation of a PROM for users of mental health services [16, 32].

The stages outlined here have been applied in part, or in entirety, in other PROM development studies [12, 16]. The framework outlined here builds upon existing guidance [5, 6] and includes additional areas for consideration particularly around dissemination.

#### Challenges of incorporating PI in PROM development

Whilst PI is recognised as beneficial and best practice, it must be acknowledged that meaningful PI is not without its challenges. There can be challenges or negative impacts that are rarely reported, however they do need to be considered in developing collaborative involvement initiatives [21]. Below we raise a variety of issues including practical, financial and ethical considerations, that may impact upon effective PI activities.

##### Need for a clear PI plan

The importance of developing a clear PI plan is essential. The research team needs to consider the remit of their project, and which stages of PROM development they will be undertaking. Discussions with PI partners will identify where PI can occur, and for what purpose. One of the first things to address is how to recruit PI partners, how many PI members to include and what skills are needed. For instance, research and interviewing experience will be required for those PI partners who undertake interviewing. It is more usual to recruit PI partners who can bring a PI perspective which reflects the research target population [13]. There may be established PI groups for the given health condition, or there may be a need to establish a new PI group, which should be as diverse as possible. Bagley et al. [33] highlight potential opportunities for PI involvement in clinical trials research within the United Kingdom. Although not exhaustive, these could also be appropriate for PROM development.

It may be that specific training is required for the research team to undertake PI activities [34], and/or training for the PI members to understand scientific aspects of PROM development. Having open discussions can highlight mentoring and support needs as well as acceptable methods of feedback. Budgeting for PI is necessary to reimburse people’s time and other expenses, but also for researcher time to engage with the PI team. In addition, there should be clarity on both sides around the expectations in terms of financial compensation and expected input. The plan should also include details of how the reporting of PI activities will occur and the extent to which the impact of PI will be evaluated.

##### Meeting and involvement practicalities

As with any research project, PI incurs a cost and time commitment of project staff. The meetings need to be carefully arranged to consider the needs of the whole PI team. Identifying those needs in advance is important to maintain good relationships within the project. Accessibility of meetings (such as location within a building, proximity to public transport, and timing), or appropriate use of technology, should be recognized and negotiated. Other aspects may include planning of comfort breaks, appropriate refreshments, awareness of caring commitments, and timely reimbursement of PI member’s time and travel costs [35, 36].

##### Preparation and delivery of PI tasks

The planning and preparation of meeting materials (such as online resources or handouts) and flexibly responding to PI input takes considerable researcher time which should be accounted for within budgets and project timescales. PROM development faces additional challenges in terms of the potential complexity of language used by researchers (e.g. psychometric terms), and practical limits imposed upon the final PROMs (e.g. length, content). How the scientific considerations of PROM development including the scope of items, the number of items, and types of appropriate items that can be included needs to be carefully explained Skills of chairing, communicating, managing difficult situations and conflicts of opinions, understanding and dealing with power relationships (both between researchers and PI members and within PI members) are different to other research skills required in outcomes research, such as those involved in advanced psychometrics, and PROMs development teams need to reflect on whether researchers have the required skills.

##### Building and sustaining relationships

It is important to build relationships where there is trust and respect, where everyone within a research team, including PI, can voice their opinion appropriately [37, 38]. Collaborative relationships such as these can take time to develop, and this will have an impact upon both the resources and timeframe of the project [39]. However, it is essential as without establishing a good partnership PI can appear tokenistic [40]. Throughout a project, there should be regular contact and communication between the PI team and the research team. By doing so it strengthens the relationship between individuals and can ensure that genuine shared decisions can occur. Sustaining long-term commitment and engagement with PI members is an ideal, providing it is wanted by all parties. There may be challenges in ensuring genuine shared decision making within the restrictions of a grant with pre-commitment to specific deliverables [41].

Within PROM development it can be very clear if PI advice is taken forward – particularly at the stage of item inclusion/reduction. Sustaining positive relationships whilst not taking forward PI advice (e.g. not including a theme that a PI member perceives to be important because it has limited relevance for some patient sub-groups) requires transparency, and time to ensure PI members understand the rational for decisions and retain a sense of ownership.

PI is often portrayed as a smooth journey – and this is not always the case. There can be frustrations on both sides [16]. PI partners may become frustrated at the formulaic way in which PROMs are developed. The team meetings themselves may be difficult: trying to ensure that progress is made whilst allowing people to voice their opinions and experiences.

Kirwan et al. [42] have developed principles on using PI in outcomes research, which focus heavily on the important area of relationship development. There is an increasing acknowledgement within institutions as to the benefits of PI within research [43, 44]. This has further been extended within some academic groups to PI partners being part of management and strategic committees. Such an inclusion has highlighted the need for positive behaviours of respect and trust, as well as identifying and addressing training needs and appropriate resources for successful PI.

##### Ethical implications, wellbeing of PI partners and appropriate commitment

It is important to recognize that PI partners are not research participants. There are no official approval requirements to initiate PI activities, such as ethics or governance approvals; however, there are some ethical considerations to consider such as mutual respect, reciprocity, shared commitment, and personal integrity [45].

As shown in the potential stages of PROM development, PI can be a substantial commitment for individuals across the whole project. The emotional wellbeing of PI participants must be maintained, and the research team should be mindful of this. There may be cases where different PI groups could inform different aspects of the PROM development to avoid overburdening a few individuals. PROM development is potentially more personal than other areas of research since it goes to the heart of understanding quality of life, and the ways in which peoples’ experiences can be less than optimal. Immersion within this reality may be emotionally demanding for PI partners. This may be particularly acute for PROM development in life-limiting conditions. Researchers can undergo training on PI facilitation to ensure appropriate interactions within team meetings.

##### Reporting of PI activities

Another challenge of PI in PROM development concerns the reporting of PI activities themselves, which recent initiatives have encouraged [46, 47]. The Guidance for Reporting Involvement of Patients and Public checklist (GRIPP) was a key driver in better reporting of PI in research studies [46]. The GRIPP2 checklist has further encouraged formal evaluation of PI activities in research. The development of the short and long form checklists both aim to improve the quality, transparency and consistency of the reporting of PI [47].

Whilst better reporting has been encouraged, academic journals do limit the number of words for article submissions (usually allowing additional online supplementary material). This places authors in the difficult position of deciding what to report as core when disseminating their research, and in some instances the role of PI within a research project may not always be fully reported. There have been pledges to support the reporting of PI. The *British Medical Journal* and its portfolio of journals have extended their requirements in reporting PI in the design, conduct and reporting of clinical studies [48]. However it is not yet clear how effective this will be, or whether allowances in word count will be altered to reflect the addition of these details. Within PROM development, project timescales and budgets relate to a specific time-limited project. However, dissemination, particularly paper writing, often occurs beyond budget timescales, creating challenges for compensated PI. Reporting of PI activities is important if the impact of PI is to be recognized by research funders.

In the reporting of PROM development researchers should clearly distinguish between patients as PI where they are co-producing the instrument versus patients as research participants. In PROM development the line may be blurred. For example, a focus group could be held to discuss instrument layout with a sample of patients as research participants or with a PI group. The content of the discussion in this case would be very similar. Pandya-Wood and colleagues [49] acknowledge that if verbal contributions arising from PI consultations are to be treated as research data then ethical approval should be sought.

##### Developing instruments for children or those with limited capacity

To date there has been little guidance on how best to incorporate PI with those under the age of 16 years as well as those with limited capacity in PROM development [50]. A recent mapping exercise explored the process of involving children and young people as research advisers. It highlighted some fundamental barriers that make meaningful involvement difficult: time, money and gatekeepers. Involvement with communities is key, and there is increasing emphasis on the role of shared decision-making in clinical care yet has been little direction on how to achieve this. Considering the potential stages of PROM development detailed in Fig. 1, it is not easy to implement PI with children at every stage. Researchers need to consider where meaningful PI can occur, and ways to suitably involve the PI team [51]. Traditional approaches may have to be replaced by innovative methods of initiating discussion, such as interactive play, learning technologies, communication aids and social media [52].

##### Developing instruments for very rare conditions

PI for very rare conditions raises issues relating to anonymity. Within a small patient community, PI members may recognize the identity of anonymised respondents via their characteristics e.g. age/gender/health or condition-stage. Furthermore, PI is likely to be inconsistent with participation in the research project, as undertaking both roles is usually avoided where possible. This is in part due to risks of affecting upon the power dynamics and sense of ownership of research findings, and undermining a clear methodology for sampling and recruitment, however, it may be beneficial in some circumstances [53].

##### The use of PI in international PROM development

Developing a single PROM which is valid across different languages, cultures and countries raises its own challenges, including locally driven concerns and interpretations of concepts [54]. Including PI within international PROM development cannot overcome these inherent difficulties. A potential tension also exists between PI as research partners in one local context, and advising on the research methods, and final PROM, versus achieving consistency in methods and outcome across different areas. Working in international collaboration may present additional considerations for PI groups including coordination and potential compromise where consistency in methods is deemed necessary. However, the need for local PI involvement is necessary to avoid research being applied to different context without appropriate adaptation.

##### Developing instruments for use in preference-based measures

Some PROMs can be used to generate Quality Adjusted Life Years used in economic evaluations of treatments [55]. Such instruments require the health states described by the PROM to be valued based on preferences of the public or patients using preference-elicitation tasks. The methods undertaken to obtain patient or public preferences are in themselves complex, and as such they bring additional requirements (or limitations) for a PROM. The PROM cannot be too long, must be completed in its entirety (i.e. there needs to be no missing values or non-relevant questions), and it is restricted to items which will work well within the preference-elicitation tasks. It adds a further complexity to PROM development that would need careful communication to the PI group.

##### Ensuring optimum PI within PRO development

There is often pressure on researchers and funders of research to undertake and complete research efficiently. Therefore optimizing PI within PROM development will be essential when considering the timescales of a project. There may be concerns regarding the investment in particular components of PI in the PROM development process. Groene [56] discuss the complexity of interpreting some psychometric data used in the development of PROMs, and the extensive training costs required for meaningful PI in this area [57]. The balance between investments in PI members versus benefit to the PROM development may differ across projects and the stages of development. Consideration should also be made to which stages PI occurs. For instance, it is possible to have different PI groups for different stages to try and minimise burden. It must be remembered that for each new PI group, there may have to be training or de-briefing of previous stages of the PROM development.

## Conclusion

We present an emerging framework identifying ways in which PI can have a meaningful role and contribution to the co-development of PROMs. Incorporating PI is an important part of the development process, and its inclusion contributes to strengthening the relevance, acceptability and validity of the PROM itself. The framework is not prescriptive as the sequence of PROM development is not uniform. The type and level of PI will vary between studies. As with PI in general, the earlier that PI can be initiated, the more scope there will be for the PROM to reflect the concerns of the target group and be ethically acceptable. It should be acknowledged that the stages outlined here are based upon our own experiences as instrument developers. We provide a figure to act as a prompt of issues to consider when developing PROMs, so that researchers can use this as a quick reference point of areas to consider. The emerging framework is a response to requests for clarity, guidance and consensus on PI in PROM development and provides a contribution to the ongoing dialogue.

## Data Availability

Not applicable.

## Abbreviations

FDA: Food and Drug Administration
GRIPP: Guidance for Reporting Involvement of Patients and Public
PI: Public Involvement
PPI: Patient and Public Involvement
PROM: Patient Reported Outcome Measure
QoL: Quality of Life
UK: United Kingdom
